# Combined genetic effects of *EGLN1* and *VWF* modulate thrombotic outcome in hypoxia revealed by Ayurgenomics approach

**DOI:** 10.1186/s12967-015-0542-9

**Published:** 2015-06-06

**Authors:** Shilpi Aggarwal, Atish Gheware, Anurag Agrawal, Saurabh Ghosh, Bhavana Prasher, Mitali Mukerji, Indian Genome Variation Consortium

**Affiliations:** Genomics and Molecular Medicine, CSIR-Institute of Genomics and Integrative Biology, Sukhdev Vihar, Mathura Road, New Delhi, India; CSIR’s Ayurgenomics Unit–TRISUTRA (Translational Research and Innovative Science ThRough Ayurgenomics), CSIR-Institute of Genomics and Integrative Biology, Sukhdev Vihar, Mathura Road, New Delhi, 110 020 India; Indian Statistical Institute, Kolkata, India; Academy of Scientific and Innovative Research (AcSIR), New Delhi, India

**Keywords:** Endophenotypes, *Prakriti*, High altitude, Deep vein thrombosis, Ayurveda, Bleeding, rs1063856, rs480902, PHD2, Predictive medicine

## Abstract

**Background:**

Extreme constitution “*Prakriti*” types of Ayurveda exhibit systemic physiological attributes. Our earlier genetic study has revealed differences in *EGLN1*, key modulator of hypoxia axis between *Prakriti* types. This was associated with differences in high altitude adaptation and susceptibility to high altitude pulmonary edema (HAPE). In this study we investigate other molecular differences that contribute to systemic attributes of *Prakriti* that would be relevant in predictive marker discovery.

**Methods:**

Genotyping of 96 individuals of the earlier cohort was carried out in a panel of 2,800 common genic SNPs represented in Indian Genomic Variation Consortium (IGVC) panel from 24 diverse populations. Frequency distribution patterns of *Prakriti* differentiating variations (FDR correction P < 0.05) was studied in IGVC and 55 global populations (HGDP–CEPH) panels. Genotypic interactions between *VWF*, identified from the present analysis, and *EGLN1* was analyzed using multinomial logistic regression in *Prakriti* and Indian populations from contrasting altitudes. Spearman’s Rank correlation was used to study this genotypic interaction with respect to altitude in HGDP–CEPH panel. Validation of functional link between *EGLN1* and *VWF* was carried out in a mouse model using chemical inhibition and siRNA studies.

**Result:**

Significant differences in allele frequencies were observed in seven genes (*SPTA1, VWF, OLR1, UCP2, OR6K3, LEPR,* and *OR10Z1*) after FDR correction (P < 0.05). A non synonymous variation (C/T, rs1063856) associated with thrombosis/bleeding susceptibility respectively, differed significantly between Kapha (C-allele) and Pitta (T-allele) constitution types. A combination of derived *EGLN1* allele (HAPE associated) and ancestral *VWF* allele (thrombosis associated) was significantly high in Kapha group compared to Pitta (p < 10^–5^). The combination of risk-associated Kapha alleles was nearly absent in natives of high altitude. Inhibition of EGLN1 using (DHB) and an *EGLN1* specific siRNA in a mouse model lead to a marked increase in vWF levels as well as pro-thrombotic phenotype viz. reduced bleeding time and enhanced platelet count and activation.

**Conclusion:**

We demonstrate for the first time a genetic link between *EGLN1* and *VWF* in a constitution specific manner which could modulate thrombosis/bleeding susceptibility and outcomes of hypoxia. Integration of *Prakriti* in population stratification may help assemble common variations in key physiological axes that confers differences in disease occurrence and patho-phenotypic outcomes.

**Electronic supplementary material:**

The online version of this article (doi:10.1186/s12967-015-0542-9) contains supplementary material, which is available to authorized users.

## Background

There has been a deluge of variations from complete human genome sequencing projects [1, 2]. Majority of the common variations are shared across world populations and contribute to 98% inter-individual variance within populations [3–6]. Identification of meaningful variations that connect to human phenotypes and explain inter-individual variability in adaptation and susceptibility differences is the current challenge [7–10]. Integrative analysis of global variation data with environmental, geographical, dietary and cultural practices across diverse populations have helped identify common variations linked to local adaptations in different eco-clines like latitude, climate as well as mode of subsistence [11–16]. These variations correlate with phenotypic, physiological and metabolic traits and overlap with signals from GWAS studies [8, 14–17]. Besides, system biology and data intensive approaches like PheWAS are being evolved to identify phenotypic links through shared genotypes [18–21]. There is a need to evolve methods to stratify individuals even within a genetically homogeneous population on the basis of their shared physiological attributes that could give rise to different health trajectories.

India has a 3,500 year old system of medicine, Ayurveda which has at its basic tenet a predictive and participatory approach for preventive and personalized medicine [10, 22]. In this approach healthy individuals are phenotypically stratified into seven different constitution types called *Prakriti* which also determines an individual’s differential susceptibility to disease, response to diet, environment and therapy [10, 22]. A unique aspect of this approach is that different phenotypic attributes of an individual, such as morphological features, skin type, physiology, metabolism, mental aptitudes, sensory perception etc. are comprehensively assessed for defining each constitution type [22]. Inter-individual variability in underlying physiological parameters related to different body tissues (qualitative and quantitative) have also been described. For instance, blood characteristics including color and viscosity are described to vary among different *Prakriti* as well as in response to geo-climatic conditions including seasonal variations. This also sets a prelude for differential susceptibility to blood related disorders like bleeding, hyper-coagulability or thrombosis. This forms the basis for personalized health maintenance and preventive medicine through diet and life style modifications (Additional file 1).

Amongst the seven types, *Vata* (V), *Pitta* (P) and *Kapha* (K) are the three phenotypic extremes that are readily distinguishable [22–25]. Many of the phenotypic features that distinguish the predominant *Prakriti* types overlap with attributes described for human adaptations. We have earlier provided molecular and genomic evidence of differences between contrasting constitution types from a genetically homogeneous background [22]. Common variations from a subset of differentially expressed genes also partitioned differently between the phenotypically stratified *Prakriti* groups [26]. Once these groups were pooled, the variations assumed an average frequency similar to the genetic background of the population. Further analysis of *EGLN1* variation helped identify, hypoxia as one of the axis and capture genetic marker attributable to specific constitutions for high altitude hypoxic adaptation [26].

We hypothesize that integration of the comprehensive phenotyping method of Ayurveda with genomics might provide scaffolds to connect major axes of variation to an individual’s phenome. In this study we carried out our analysis on a set of ~2,800 polymorphic genic SNPs, represented in the Indian Genome Variation Consortium panel [27, 28]. We identify common variations that differ between healthy individuals of contrasting *Prakriti* types in genes that govern blood cell traits, hemostasis, metabolism, lipid homeostasis, etc. We could thread hypoxia (*EGLN1*) to hemostasis (*VWF*) and red blood cell traits (*SPTA1*) in a constitution specific manner. We provide genetic and experimental evidence of how in the background of earlier reported *EGLN1* variations that link to differences in hypoxia responsiveness, vWF variations/levels could modulate risk for thrombosis. We thus propose that an Ayurgenomics approach could enable assembly of common variations linked to different physiological axes which could assume importance in adaptability to different geo-climatic conditions and susceptibility to diseases.

## Methods

### Study subjects

The study was carried out on the same samples [22, 26] that have been used in our earlier studies. Sample collection was carried out following approval of Institutional Bioethics Committee (IBC). Briefly the samples comprised of (1) 96 individuals comprising of extreme constitution types *Vata* (n = 39) *Pitta* (n = 29) and *Kapha* (n = 28) identified from an initial phenotyping of 850 volunteers on the basis of Ayurveda methods and recruited in our earlier study. (2) 552 samples from 24 diverse Indian populations from the existing panel of IGVC [26, 28]. These include 92 heterogeneous phenotype controls (IE-pool) from Indo-European North Indian large populations (size >10 million).

### Genetic study and analysis

We carried out our analysis on a panel of 2,800 SNPs from 776 genes on whom the genotype information was available from the IGVC panel Phase II (http://igvbrowser.igib.res.in, [29]). These SNPs were genotyped in VPK samples using Illumina Bead Array platform. The SNPs tagged a set of representative genes/genic regions that have been implicated in a number of monogenic and complex diseases. These genes mapped to diverse biological processes and molecular functions. Genotype data on *EGLN1* and *VWF* from 55 populations were retrieved from the data of Stanford Human Genome Diversity Panel (HGDP) SNP Genotyping Data on 650 K Illumina arrays available on HGDP selection browser (http://hgdp.uchicago.edu/, [30]). The altitude of each of the populations in the HGDP panel was retrieved from the earlier study [31].

Fisher’s exact test [32] was carried out for testing genotypic and allelic associations between the *Prakriti* types as well as between *Prakriti* and IE-pool background. Correction for multiple testing was done using the FDR method.

We compared the frequency of combined genotypes of *EGLN1* and *VWF* between the *Prakriti* types and in high altitude natives and a related population from sea level (Additional files 2, 3). Combined genetic analysis was carried out considering *EGLN1* allele associated with high altitude adaptation and *VWF* allele associated with non thrombotic phenotype as protective alleles. We evolved a scoring mechanism wherein the total count of the protective alleles in *EGLN1* and the *VWF* genotypes in an individual was calculated. Thus, a score of “0” corresponds to individuals homozygosity for the risk allele ‘C’ at both rs480902/rs1063856 (that is, the genotype CC/CC) and a score of 4 where all the alleles were protective (that is, the genotype TT/TT). All the nine genotypes were considered and converted to these scores. Multinomial logistic regression was performed to associate these scores and the *Prakriti* types (K/V/P). We used binary logistic regression to test for differences in the distribution of scores between (1) individuals residing at high altitude and those residing at sea-level and (2) individuals residing at high altitudes and the *Prakriti* group “P”. In order to study the possible relationship between the scores of individuals belonging to the different HGDP populations and the altitudes corresponding to these populations, we computed the Spearman’s rank correlation between the mean scores of each population and the altitudes (Additional file 4).

### Animals and experimental protocol

Male BALB/c mice (8–10 week old with an average weight of 25 grams; obtained from CSIR-Central Drug Research Institute, Lucknow, India) were acclimatized for 1 week before starting the experiments. All animals were maintained as per the guidelines of the Committee for the Purpose of Control and Supervision of Experiments on Animals (CPCSEA), and the protocols were approved by the Institutional Animal Ethics Committee of CSIR-IGIB.

#### Ethyl-3, 4-dihydroxy benzoic acid (DHB) treatment

Mice were divided into three groups (*n* = 5–6), according to the treatment: Vehicle (10% ethanol), D5 (DHB 5 mg/kg), D10 (DHB 10 mg/kg). DHB (TCI AMERICA, Portland, OR, USA) and 10% ethanol was administered via Intraperitoneal (I.P.) injection at 100 µl volume for 7 consecutive days.

#### siRNA treatment

Mice were divided into three groups (n = 5–6), according to the treatment: Naive (untreated mice), Scram siRNA (scrambled siRNA treated mice) and PHD2 siRNA (PHD2 siRNA treated mice). For assessing the systemic effect we used the intranasal route for siRNA delivery as described previously [31]. Briefly, 90 µg of PHD2 (Dharmacon, Lafayette, CO) or scrambled siRNA (Dharmacon, Lafayette, CO) dissolved in 30 ml of ultrapure DNase and RNAse free water was administered intranasally to isoflurane-anesthetized mice. SiRNA was given on day 1, 3 and 5. The animals were sacrificed on day 6 from the start of the experiment.

### Tail bleeding assay (bleeding time assay)

On the final day of protocol (DHB or siRNA experiment), mice were anaesthetized in prone position. A 10 mm segment of the tail from the distal end was amputated with sharp scalpel. The tail was then immediately immersed in 50 ml of PBS (pre-warmed to 37°C in a water bath) contained in a Falcon tube. The position of tail was kept vertical with tip positioned about 2 cm below the PBS meniscus. The bleeding time was then determined by monitoring the duration of animal tail bleeding until it ceased, using a stop clock.

### Biochemical analysis

Blood was obtained by cardiac puncture and collected in EDTA coated MiniCollect tubes (Greiner Bio-One Gmbh, kremsmünster, Austria). Plasma was isolated by centrifugation of blood at 1,000*g* for 15 min at 4°C and kept in −80°C for further use. HIF1α and vWF levels were measured in plasma sample by using ELISA kit (USCNk, Wuhan, China) as per the manufacturer’s protocol. The whole blood was used to measure the total count of platelets and its distribution width by using automated hematology analyzer (Nihon kohden, Japan). Assessment of active and total Platelet count was carried out through flow cytometry using FACSCalibur (BD Biosciences, USA). Briefly, 100 μl of blood was diluted in 400 μl of TBS. This diluted whole blood (1:4) was then incubated with APC conjugated anti-CD62P (eBioscience Inc, San Diego, CA, USA) and FITC conjugated anti-CD41 (eBioscience Inc, San Diego, CA, USA) for 15 min. Matched fluorescein conjugated isotype control antibodies were used simultaneously for staining, to set the threshold and exclude nonspecific binding. Activity was compared using CellQuest Pro software (BD Biosciences, USA).

### Statistical analysis

Statistical significance was determined by the Student’s t test. Non parametric statistical test Mann–Whitney rank sum test or Wilcoxon matched pair test was used wherever the data does not follow the Gaussian distribution. Analysis was done using GraphPad Prism software 5.0.

## Results

### Common variations partition differently amongst healthy individuals of extreme constitution types

**Figure 1:**
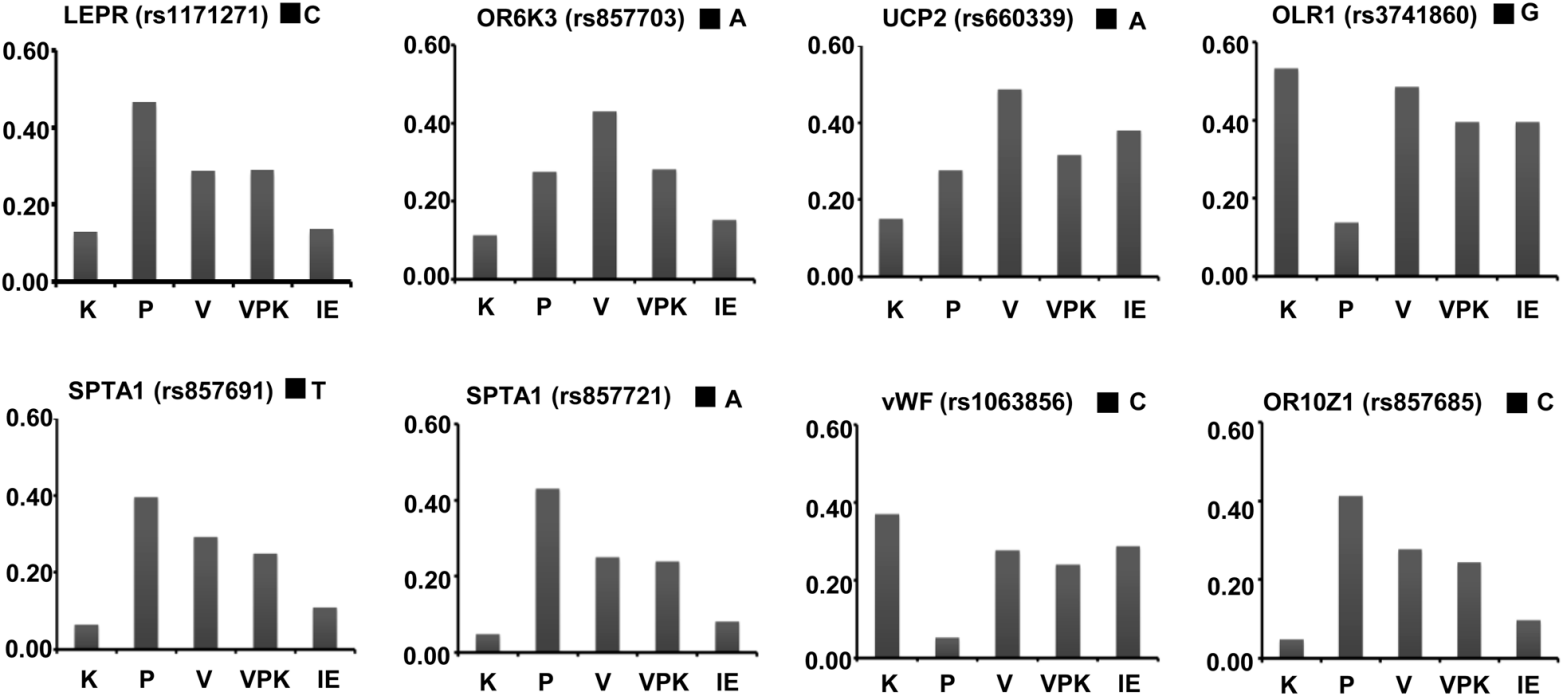
Representation of allele frequencies of common variations amongst extreme constitution types. A representative set of SNPs that show significant difference between the constitution types *Kapha* (K), *Pitta* (P), *Vata* (*V*) and differ from the VPK/IE pool are depicted. The gene and SNP with the alleles are given in each panel. *IE* represents individuals with heterogeneous phenotypes from Indo-European populations and *VPK* represents individuals of different constitution types pooled into a single group.

Indian Genome Variation Consortium (Phase II) houses variation information on 2,800 tag SNPs from approximately 776 genic regions in diverse populations of India. We studied the distribution of these SNPs in *Vata* (V), *Pitta* (P) and *Kapha* (K) subgroups that were identified from our earlier study [22]. The details of recruitment and assessment of *Prakriti* types has been described earlier [22]. 92 individuals who were not phenotyped for their constitution types but were from the same ethno-genetic background namely, Indo-European, large populations were used as heterogeneous phenotype controls (IE-pool).The details of the populations are provided in methods above. We observed that nine SNPs from seven genes (*SPTA1*, *VWF*, *OLR1*, *UCP2*, *OR6K3*, *LEPR*, and *OR10Z1*) have significant allele frequency differences between the constitution types even after correction for multiple testing with false discovery rate threshold set at 5% (Figure 1; Table 1). Even though we had selected tag SNPs, two SNPs (rs857691, rs857721) of *SPTA1* were different between P and K. P seemed to be differentiated from both V and K at the *OLR1* locus (rs3741860). There was a significant difference between K and V at the *OR6K3* (rs857703) and *UCP2* (rs660339) locus. P and K besides *SPTA1* and *OLR1* also differed at the *VWF* (rs1063856) and *OR10Z1* (rs857685) locus. Most importantly, once the constitution types were pooled, these contrasting allele frequencies were averaged out and the pooled frequencies did not differ significantly from the IE-background. Further comparison of each constitution group with the IE-pool revealed significant difference (FDR correction at 5%) between P and IE with respect to *LEPR* (rs1171271), *SPTA1* (rs857691, rs857721), *OR10Z1* (rs857685) and V and IE with respect to *OR6K3* (rs857703) (Additional file 5).

**Table 1:** SNPs that show significant differences between the *Prakriti* groups after FDR correction for multiple testing set at a threshold of significance of p < 0.05

Gene	SNP	Variation	Comparison	Allele	Allele frequency 1	Allele frequency 2	P value
			Allele frequency (1 vs. 2)				
EPR	rs1171271	C/T	PvsK	C	0.46	0.13	5.40E−05
OR6K3	rs857703	A/G	KvsV	A	0.11	0.43	4.44E−05
UCP2	rs660339	A/G	KvsV	A	0.15	0.49	4.23E−05
OLR1	rs3741860	A/G	VvsP	G	0.49	0.14	2.87E−05
OLR1	rs3741860	A/G	PvsK	G	0.14	0.53	6.35E−06
SPTA1	rs857691	C/T	PvsK	T	0.40	0.06	1.63E−05
SPTA1	rs857721	A/T	PvsK	A	0.43	0.05	6.05E−07
*VWF*	rs1063856	C/T	PvsK	C	0.05	0.37	1.55E−05
OR10Z1	rs857685	A/C	PvsK	C	0.41	0.05	1.46E−06

## Additive effect of *VWF* in high altitude adaptation with *EGLN1*

**Figure 2:**
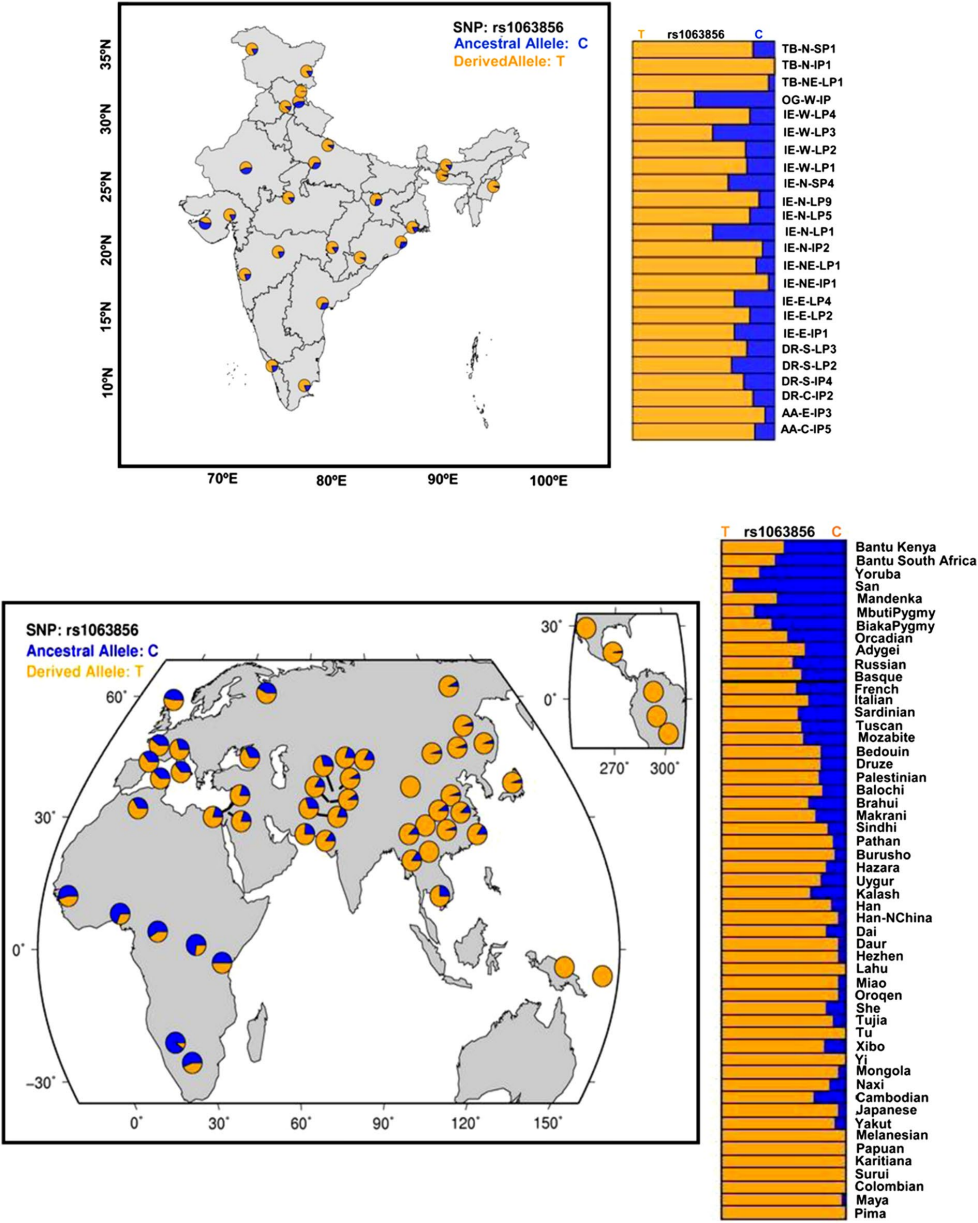
Distribution of ancestral ‘C’ allele (rs1063856) frequency of *VWF* gene associated with thrombosis in diverse IGVC and HGDP-CEPH populations. **a** Frequency in the 24 IGV populations. TB-N-IP1, a population from high altitude has fixation for the derived allele. **b** Spatial frequency map of rs1063856 in the HGDP-CEPH panel of 55 populations retrieved from HGDP selection browser. There is a clinal variation in the frequency of derived allele as populations moved out of Africa.

The frequency of the ancestral ‘C’ allele of rs1063856 in *VWF* gene, that is reported to be associated with atherosclerosis [33] was high in *Kapha* (Figure 1) whereas the derived allele was high in *PittaPrakriti*. Incidentally, the allele linked to *Pitta* was nearly fixed in a native population of Tibeto- Burman background residing at high altitude. In contrast in the Indo-European population, which was the background of these constitution types, the ancestral allele was more frequent (Figure 2). In the HGDP-CEPH population there was a clinal distribution of the ancestral ‘C’ allele (rs1063856) of *VWF* with increase in frequency of derived non-thrombotic allele in the Eastern populations (Figure 2).

Interestingly we reported a similar observation with respect to a genetic variation in *EGLN1* linked to its expression in our earlier study. We observed a variation linked to lower expression of *EGLN1* in Pitta *Prakriti* as well as natives of high altitude both in Indian and global populations whereas the other allele significantly overrepresented in Kapha and sea level sojourners who developed high altitude pulmonary edema [26]. However in case of *EGLN1* it was ancestral allele that was fixed in natives of high altitude whereas in case of *VWF* we observed the derived allele linked to low levels of vWF to be nearly fixed in high landers. We hypothesize that low levels of *EGLN1* due to environmental hypoxia or a genetic variation could result in elevated vWF as a consequence of angiogenesis and confer thrombosis risk. Hence we anticipate, fixation of a non-thrombotic/derived allele in the background of protective *EGLN1* genotype could give an advantage in high altitude condition as well as in conditions where HIF related pathways are expected to be elevated.

**Figure 3:**
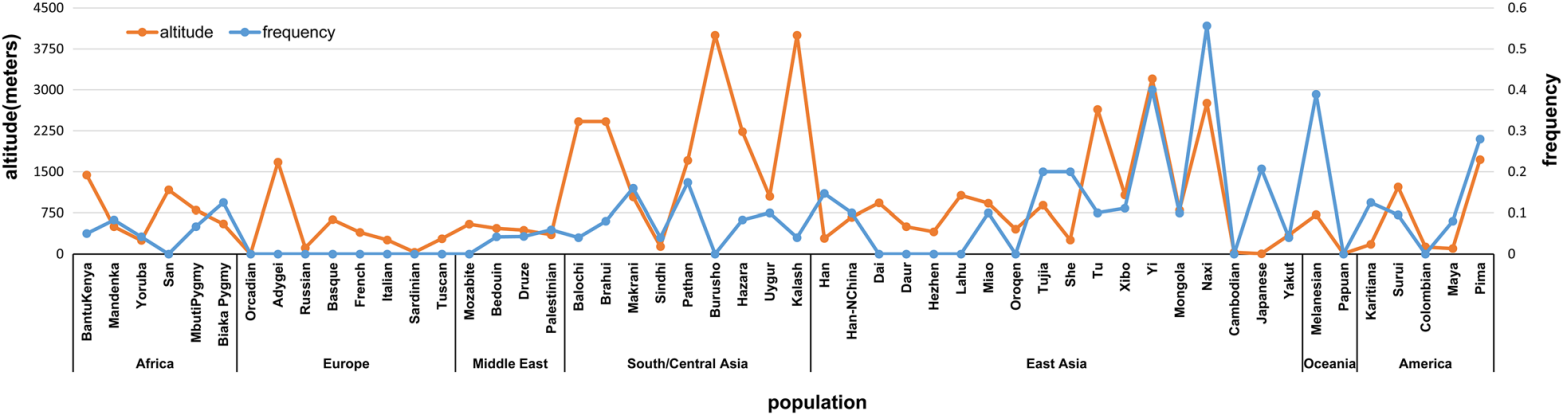
Distribution of frequency of homozygous genotype scores (4) for protective alleles at both loci *EGLN1.* rs480902 and *VWF* rs1063856 in diverse HGDP-CEPH populations from different altitudes. Diverse populations especially from East Asia residing at high altitude selectively retain the combination of ancestral allele of *EGLN1* and derived allele of *VWF.*

In order to test the above hypothesis, we compared the frequency of combined genotypes of *EGLN1* and *VWF* between the *Prakriti* types and also compared their presence in high altitude natives and a related population from sea level as described in the methods above. *Pitta* differed significantly from *Kapha* (p value 7.4 × 10^−5^) and *Vata* (p value 0.013) with respect to the additive score of the protective genotypes. This difference was also observed between the high altitude natives and their genetic counterpart from the lowland regions (p value 7.18 × 10^−5^). Both *Pitta* and high altitude natives had the genotype in *EGLN1* that was HA associated and *VWF* that was linked to protection from thrombosis. This implies a physiological link between the hypoxia and hemostasis axes through genetic interactions between *EGLN1* and *VWF*. The mean score of the protective genotype also demonstrated a significant correlation with altitude (p value 0.009) in the HGDP–CEPH populations. This correlation was more strong in East Asian populations from the HGDP panel. Incidentally the high altitude population from India also belongs to the same genetic background (Figure 3).

## Validation of a functional link between *EGLN1* and *VWF* in mouse model

**Figure 4:**
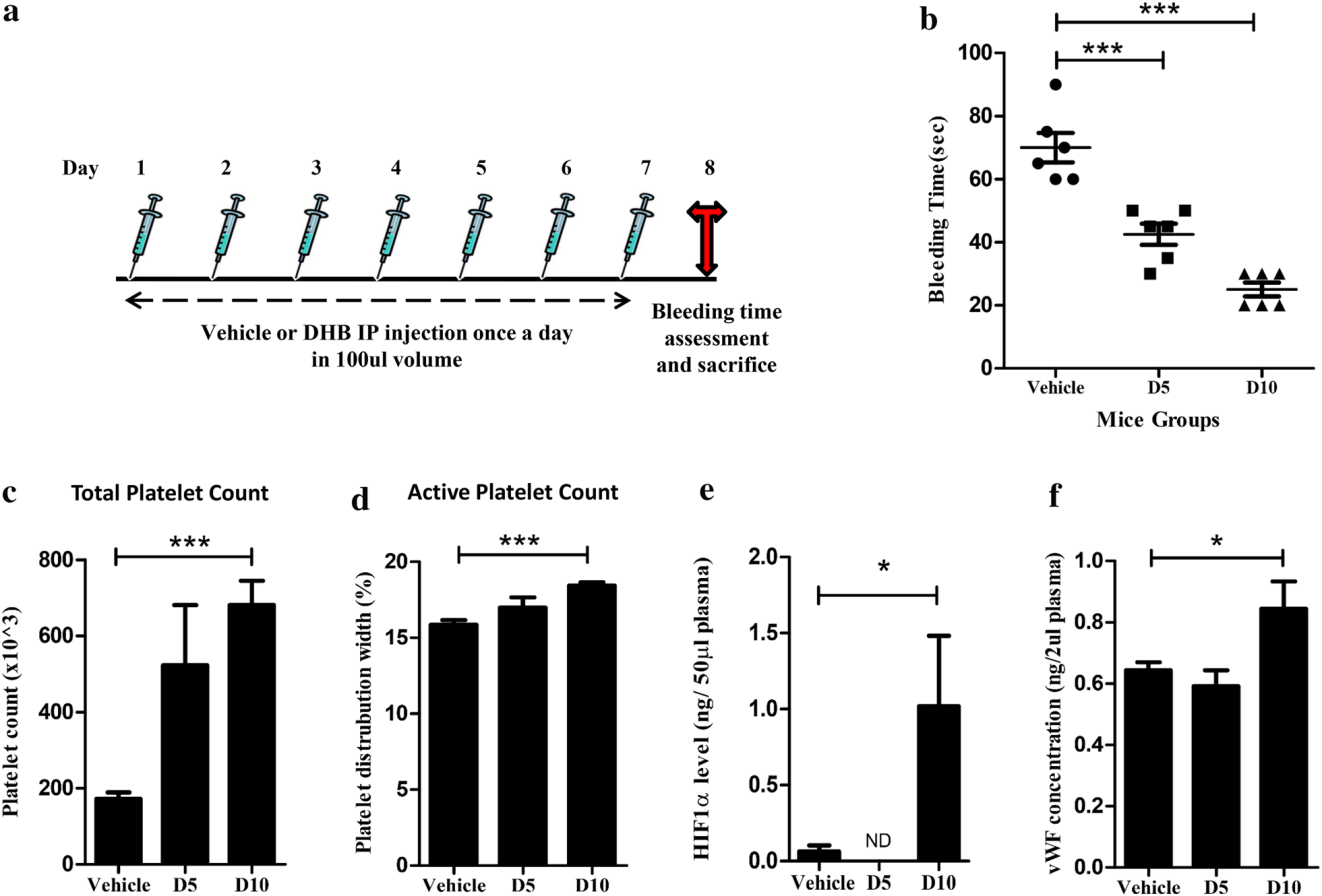
Effect of chemical inhibition of PHD or EGLN in mouse on various blood parameters. **a** Schematic representation of DHB experiment protocol. PHD inhibition was done by giving DHB (ethyl-3, 4, dihydroxybenzoic acid) treatment in mice. DHB was administered from day 1 to 7. On day 8 mice tail bleeding assay was performed. **b** Decrease in tail bleeding time of mice treated with DHB in a dose dependent manner. **c**, **d** PHD inhibition in mice by chemical DHB treatment leads to increase in total platelet count and its distribution width (considered here as active platelet parameter). Both parameters were assessed through automatic hematology analyzer in mouse whole blood (anticoagulated) as described in methods. **e**, **f** ELISA for estimation of HIF-1α and *VWF* antigen levels in mouse plasma after PHD inhibition. *ND* not detectable. Data shown as mean ± SEM. *p ≤ 0.05, ***p ≤ 0.001 (n = 6 per group).

**Figure 5:**
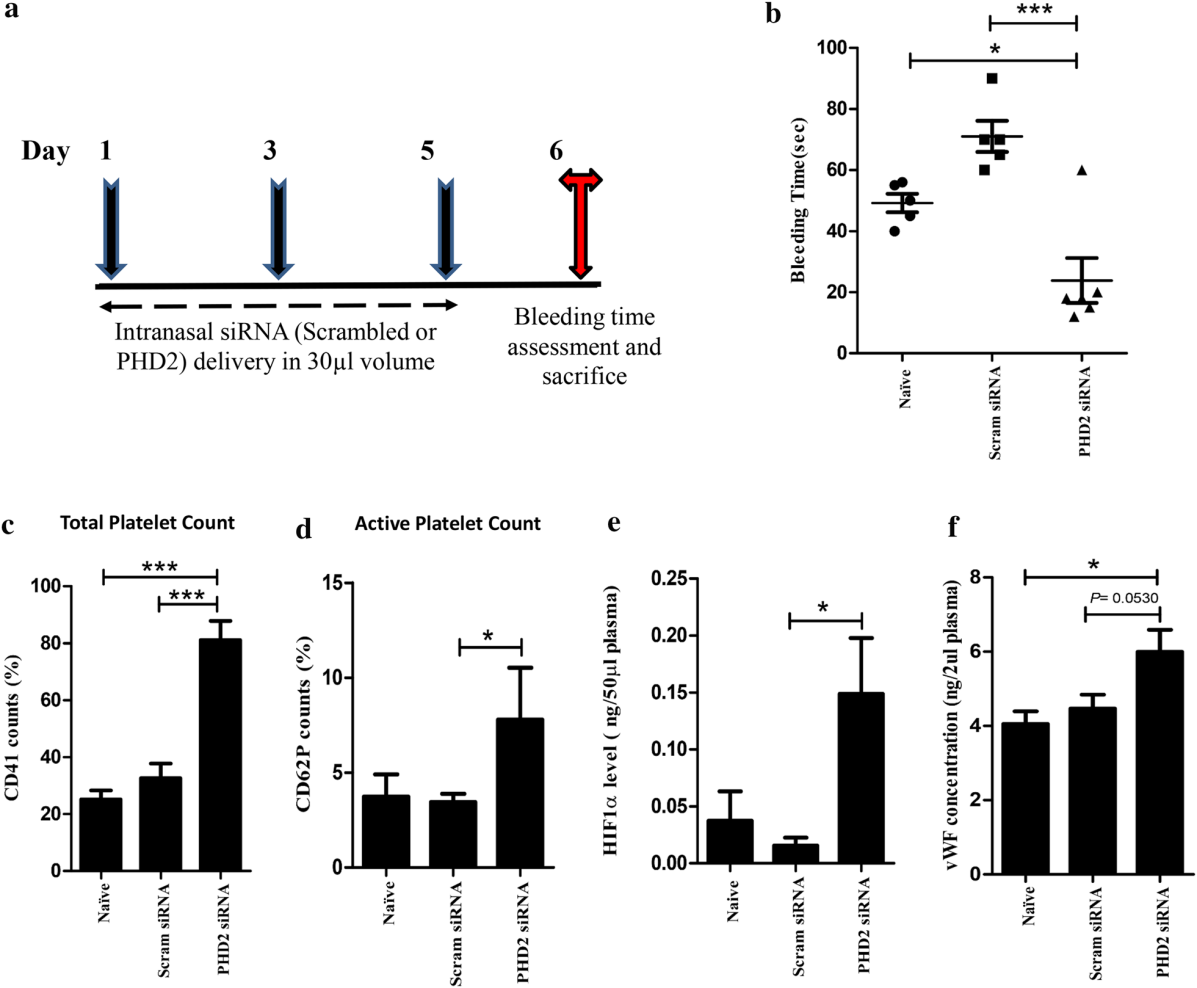
Knockdown of PHD2 by siRNA in mice leads to pro-thrombotic phenotype. **a** Schematic representation of siRNA experiment protocol. Scrambled or PHD2 siRNA was given intra-nasally to mice on day 1, 3 and 5. On day 6 mice were subjected to tail bleeding assay. **b** Tail bleeding time (in seconds) measured in mice treated with siRNA (Scrambled or PHD2) or in untreated (naïve) mice groups as described in methods. **c**, **d** Total and active platelet count assessed in mouse whole blood via flow cytometry. CD62P and CD-41 antibody were used to label total and active platelets respectively **e**, **f** ELISA for estimation of *VWF* and HIF-1α antigen levels in mouse plasma. Data shown as mean ± SEM. *p ≤ 0.05, ***p ≤ 0.001 (n = 5–6 per group).

We carried out a chemical inhibition study in mouse model to validate whether hypoxia condition confers an elevated risk for thrombosis through the *EGLN1* axes. PHD (EGLN) inhibition was induced by Ethyl-3-4-dihydroxybenzoic acid (DHB) a general PHD inhibitor, in male BALB/c mice in two different doses. DHB was administered from day 1 to 7 and animals were sacrificed on day 8 and bleeding time assay was performed. Other blood parameters were also measured on the same day. A dose dependent decrease in mouse tail bleeding time was observed after PHD2 inhibition. Treatment with DHB also significantly increased the total platelet count and its distribution width. Further, high dose of DHB increases the plasma *VWF* antigen levels (Figure 4). In order to address the question whether chemical inhibition of PHD was specific we tested the effect by specific siRNA treatment against PHD2 in mice. We observed PHD2 siRNA treatment to mice leads to significant decrease in tail bleeding time (Figure 5). In addition, siRNA knockdown of PHD2 lead to an increase in total platelet count drastically in mice blood. It also increased HIF-1α levels in plasma sample of mice (Figure 5). To confirm whether this observed effect is due to vWF, we checked the levels of vWF in plasma. We found that the PHD2 siRNA treatment causes an increase in vWF levels, which is border line significant (p < 0.0530) (Figure 5).

## Discussion

We studied the distribution of common variations, represented in the IGVC panel, in the healthy individuals of extreme Ayurvedic constitution types. These predominant constitutions types belong to one of the genetically homogeneous clusters identified in the IGVC project that comprises Indo-European population from North India [27, 28]. We observed significant differences in SNP allele frequency in seven genes (*SPTA1*, *VWF*, *OLR1*, *UCP2*, *OR6K3*, *LEPR*, and *OR10Z1)* between the constitution types. These genes are important players in governing major axes like blood cell traits, hemostasis, metabolism and lipid homeostasis in healthy individuals and have also been associated with risk for bleeding disorders, CVD, stroke, obesity, atherosclerosis [34–42]. These not only differ between constitution types but also correlate with phenotypes that differentiate the *Prakriti* types. *Prakriti* as mentioned above not only exhibit phenotypic differences but also renders differential responsiveness to environmental conditions as well as susceptibility to diseases. Extreme *Prakriti* types in any population are at the end of phenotypic spectrum and have contrasting responsiveness to intrinsic and extrinsic stimuli. Majority of the variations that differ between the extreme *Prakriti* types are shared across diverse populations and their frequencies can vary on account of selection pressures such as climatic conditions, mode of subsistence, pathogen pressure and cultural practices even in genetically related populations [12–14], (Additional file 6). Many of these also overlap with signals associated with different diseases and phenotypic traits in GWAS studies [35–42]. Thus the same variation associated with a constitution type could assume importance in adaptation or disease susceptibility/protection based on the context.

The ancestral ‘C’ allele of *VWF* rs1063856 has been associated with elevated plasma von Willebrand factor levels and the risk of incident venous thrombosis [43, 44]. Besides mutation in *VWF* gene leading to its reduced expression has also been linked to bleeding susceptibility [33].

In an earlier study, we have reported expression and allelic frequency differences in an oxygen sensor *EGLN1* between *Pitta* and *KaphaPrakriti* types [26]. The genotype linked to higher expression of *EGLN1* was observed in *Kapha* and HAPE. EGLN1 inactivates HIF-1a in high oxygen conditions. Under hypoxic conditions EGLN1 is inactive leading to elevated HIF-1a levels, which switches on a large number of genes including those linked to angiogenesis [45, 46]. The same variation in *EGLN1* gene has been associated with high altitude adaptation in multiple populations of the world [47–49]. Levels of vWF have been shown to be elevated in cellular hypoxic conditions [50–53].

It is conceivable that in the background of an ancestral favourable allele, there might be selection for newer alleles that are physiologically advantageous. For instance, in the presence of an ancestral allele of *EGLN1* that favours high altitude adaptation, elevated risk for thrombosis can arise as a consequence of angiogenesis due to high levels of vWF. We could functionally validate the observed link obtained between *EGLN1* and *VWF* genotypes through chemical and siRNA mediated inhibition of PHD2 in a mouse model (Figures 4, 5). In the presence of hypoxia as a consequence of PHD2 inhibition not only *VWF* but markers that are associated with increased risk for thrombosis were observed to be elevated. Our results support the previous findings [54–56]. Thus increasing concentration of the chemical inhibitor DHB which inhibits the activity of PHD2 leading to enhanced hypoxic response results in a prothrombotic phenotype viz increased platelet count and activation and reduced bleeding time. This is in line with previous work showing that hypoxia elevates the *VWF* expression [50–53]. Thus, both chemical and siRNA mediated inhibition of PHD2 leads to a pro-thrombotic phenotype in mice corroborating with our genetic finding.

Therefore, presence of a derived (non-thrombotic) allele of *VWF* in the background of elevated HIF could confer protection from thrombosis in *PittaPrakriti* types as well as in high altitude natives.

The ancestral allele linked to higher level of *VWF* needed for coagulation might be important in protection from blood loss due to various reasons. However, this might not be favourable in high altitude where already a constitutive state of hypoxia can promote thrombosis. This could be the reason for near fixation of the derived non-thrombotic state of *VWF* in high altitude population of India as well as in the other high altitude regions of the world. Incidentally the high altitude region in India where TB-N-IP1 resides is also classified as a dry desert. Prolonged low oxygen conditions and dryness are known to precipitate deep vein thrombosis through platelet aggregation for instance in long distance fliers. Our observation might also assume importance in identifying individuals who might be differentially susceptible to deep vein thrombosis.

In Ayurveda, seven different constitutions types with varying proportions of *Vata*, *Pitta* and *Kapha* are described to be present in all populations. These proportions are determined by ethnicity, heritability, geo-climatic adaptation, time and season which also govern the relative phenotypic optimum of the population. *Prakriti* assessment is carried out keeping these aspects in context for an individual. *Prakriti* integrates underlying physiological variability, adaptation to different climatic conditions as well as susceptibility to diseases. An environment such as high altitude which is described as *Kapha*-*Vata* promoting can lead to elevated *Pitta* component as a part of adaptation. However, amongst the sojourners from sea level who are not naturally adapted, individuals who have elevated *Pitta* levels might be more protected from high altitude sickness. This might explain the sharing of genotypes between high altitude natives and *Pitta* where adaptation might be a cause in the earlier one and in the later it can confer adaptability to high altitude environment. This observation lends credence to the Ayurveda’s personalized recommendation of diet and life style based on an individual’s constitution, habitat, seasons etc.

## Conclusion

In this study, we demonstrate how stratification of normal healthy individuals can help uncover a novel and non-intuitive genetic link between hypoxia and hemostasis axes that is important both in adaptation and disease susceptibility. Complementation of Ayurveda based phenotypic classification of healthy individuals with population, environmental and geographical genomics approaches might not only allow us to partition common variations among healthy individuals but also help connect key physiological axes that confers differences in disease occurrence and patho-phenotypic outcomes. Given that *Prakriti* assessment is important in personalized recommendations for health and disease management, this could be important for translational medicine.

## Additional files

Additional file 1: Original Sanskrit versions supporting the text. Additional file 2: Total scores of protective alleles in combined genotypes of *VWF* and *EGLN1* in *Prakriti* types. Additional file 3: Total scores of protective alleles in combined genotypes of *VWF* and *EGLN1* in high altitude and a genetically related low altitude population in India. Additional file 4: Total scores of protective alleles in combined genotypes of *VWF* and *EGLN1* in CEPH–HGDP population. Additional file 5: Allele frequency differences between *Prakriti* types and IE pool. Additional file 6: Association of SNPs varying between *Prakriti* with selection pressures such as climatic conditions, mode of subsistence, pathogen pressure and cultural practices (source: http://genapps2.uchicago.edu:8081/dbcline/).
