# Supplementary material for: Combined genetic effects of EGLN1 and VWF modulate thrombotic outcome in hypoxia revealed by Ayurgenomics approach
Source: J Transl Med. 2015 Jun 6;13:184. doi: 10.1186/s12967-015-0542-9 (PMC4457985; doi:10.1186/s12967-015-0542-9)
Supplement: Additional file 1: — Original Sanskrit versions supporting the text. [file 12967_2015_542_MOESM1_ESM.pdf]

Ayurveda descriptions about Blood characteristics, hemopoeisis, and inter- individual variability associated with it

Effect of lifestyle and dietary practices as well as prolonged exposure to a geo-climatic condition and seasonal variations on hemopoeisis

विधिना शोणितं जातं शुद्धं भवति देहिनाम् ।
देशकालौकसात्म्यानां विधिर्यः संप्रकाशितः ।।३।।-
C.Su.24/3

सात्म्यशब्दस्य देशादिभिः प्रत्येकमन्वयः| ओकसात्म्यम् अभ्याससात्म्यम्
Chakrapani commentary on C.Su.24/3

Effect of time, season and weather conditions on blood characteristics

Hypercoagulability in cold and cloudy weather conditions

तत्र, दुर्दिने दुर्विद्धे शीतवातयोरस्विन्ने भुक्तमात्रे स्कन्दत्वाच्छोणितं न स्रवत्यल्पं वा स्रवति ।।२७।। - Su. Su.14

दुर्दिने इति मेघाच्छादितेऽह्नि|

अनृतौ दुर्दिने चेत्यादौ शीतदोषानित्यनेन शीतकाले

Chakrapani on Su.Su.14/27

शीतवातयोरिति कालकृतो दोषः Dalhana on Su.Su. 14/27

Characteristics of blood with respect to colour

तपनीयेन्द्रगोपाभं पद्मालक्तकसन्निभम् ।
गुञ्जाफलसवर्णं च विशुध्दं विध्दि शोणितम् ।।२२।। - **C.su. 24**

Inter-individual variability in normal color of blood

विशुद्धरक्तलिङ्गे नानावर्णता वातादिप्रकृतित्वान्मनुष्याणाम्

Chakrapani commentary on C.Su.24/22

Physiological role of Blood described in Ayurveda
Health, Immunity, skin complexion, carrier of vital breath (oxygen carrying), and longevity

तद्विशुद्धं हि रुधिरं बलवर्णसुखायुषा ।
युनक्ति प्राणिनं प्राणः शोणितं ह्यनुवर्तते ।।४।। -**C.su. 24**

Altered characteristics of blood due to vitiation by Vata, Pitta and Kapha

अरुणाभं भवेद्वाताद्विशदं फेनिलं तनु ।
पित्तात् पीतासितं रक्तं स्त्यायत्यौष्ण्याच्चिरेण च ।।२०।।
prolonged bleeding time

ईषत्पाण्डु कफाद्दुष्टं पिच्छिलं तन्तुमद्घनम् । **thick blood**
संसृष्टलिङ्गं संसर्गात् त्रिलिङ्गं सान्निपातिकम् ।।२१।। C. Su.24

Pitta related disorders include excessive bleeding tendencies inflammation etc

पित्तविकारांश्चत्वारिंशतमत ऊर्ध्वमनुव्याख्यास्यामः- ऊष्माधिक्यं च, शोणितक्लेदश्च, मांसक्लेदश्च, त्वग्दाहश्च, (मांसदाहश्च), ------------------ रक्तपित्तं च, रक्तमण्डलानि च, जीवदानं च, तमःप्रवेशश्च, इति चत्वारिंशत्पित्त विकाराः पित्तविकाराणामपरिसंख्येयानामाविष्कृततमा व्याख्याताः ।।१४।।
- C. Su.20/14

Kapha related disorders includes obesity, cardiovascular disorders, atherosclerosis etc

श्लेष्मविकारांश्च विंशतिमत ऊर्ध्वं व्याख्यास्यामः, तद्यथा-तृप्तिश्च,
तन्द्रा च, निद्राधिक्यं च,--- स्तैमित्यं च, हृदयोपलेपश्च, कण्ठोपलेपश्च, ------
धमनीप्रति(वि)चयश्च, गलगण्डश्च, अतिस्थौल्यं च, शीताग्निता च, उदर्दश्च,
श्वेतावभासता च, श्वेतमूत्रनेत्रवर्चस्त्वं च, इति विंशतिः श्लेष्मविकाराः
श्लेष्मविकाराणामपरिसंख्येयानामा- विष्कृततमा व्याख्याता भवन्ति ।।१७।।
- C. Su.20/15

Effect of geo climatic condition on health profile of residents including their strengths, disease susceptibilities, diet, lifestyle practices etc

तत्र भूमिपरीक्षा आतुरपरिज्ञानहेतोर्वा स्यादौषधपरिज्ञानहेतोर्वा । तत्र
तावादियमातुरपरिज्ञानहेतोः । तद्यथा- अयं कस्मिन् भूमिदेशे जातः संवृद्धो
व्याधितो वा, तस्मिंश्च भूमिदेशे मनुष्याणामिदमाहारजातम्, इदं विहारजातम्,
इदमाचारजातम्, एतावच्च बलम्, एवंविधं सत्त्वम्, एवंविधं सात्म्यम्, एवंविधो
दोषः, भक्तिरियम्, इमे व्याधयः, हितमिदम्, अहितमिदमिति प्रायोग्रहणेन ।
औषधपरिज्ञानहेतोस्तु कल्पेषु भूमिपरीक्षा वक्ष्यते ।।९३।। **C.Vi.8/93**

Suitability of diet, LS, disease prevalence etc

Ayurveda assigns Prakriti to habitats based on its characteristics wherein High mountain regions are Kapha vata dominant where diseases of Kapha Vata nature are more prevalent

त्रिविधः खलु देशः-जाङ्गलः, आनूपः, साधारणश्चेति ।
अथानूपो हिन्तालतमालनारिकेलकदलीवनगहनः, -----------
क्षितिधरनिकुञ्जोपशोभितः मन्दपवना- नुवीजिताक्षितिरुहगहनः,
अनेकवनराजीपुष्पितवनगहनभूमिभागः, ------------सुकुमारपुरुषः,
पवनकफप्रायो ज्ञेयः, ---।।८।। - C.Ka. 1/8

देशस्त्वानूपो जाङ्गलः साधारण इति । तत्र, बहूदकनिम्नोन्नतनदीवर्षगहनो
मृदुशीतानिलो बहुमहापर्वतवृक्षो मृदुसुकुमारोपचितशरीरमनुष्यप्रायः
कफवातरोगभूयिष्ठश्चानूपः, ---------------- ।।४२।।

-Su.Su.35/40

Diet and life style recommendations for maintenance of health are based on individual's Prakriti, habitat, season etc

देशकालात्मगुणविपरीतानां हि कर्मणामाहारविकाराणां च क्रियोपयोगः
सम्यक्, ----------- स्वस्थवृत्तमेतावद्धातूनां साम्यानुग्रहार्थमुपदिश्यते ।।८।।

-C. Sa.6/8
