# Supplementary material for: Combined genetic effects of EGLN1 and VWF modulate thrombotic outcome in hypoxia revealed by Ayurgenomics approach
Source: J Transl Med. 2015 Jun 6;13:184. doi: 10.1186/s12967-015-0542-9 (PMC4457985; doi:10.1186/s12967-015-0542-9)
Supplement: Additional file 5: — Allele frequency differences between Prakriti types and IE pool. [file 12967_2015_542_MOESM5_ESM.doc]

Supplementary Table 1: SNPs that show allele frequency differences between a constitution type and IE pool after FDR correction for multiple testing set at a threshold of FDR < 0.05 (Fisher’s exact test)

| Gene | SNP | Variation | Comparison  allele frequency  (1 vs. 2) | Allele | Allele  frequency 1 | Allele  frequency 2 | P value |
| --- | --- | --- | --- | --- | --- | --- | --- |
| LEPR | rs1171271 | C/T | IEpoolvsP | C | 0.1359 | 0.4655 | 5.88E-07 |
| OR6K3 | rs857703 | A/G | IEpoolvsV | A | 0.1522 | 0.4306 | 5.40E-06 |
| SPTA1 | rs857691 | C/T | IEpoolvsP | T | 0.1087 | 0.3966 | 4.93E-06 |
| SPTA1 | rs857721 | A/T | IEpoolvsP | A | 0.08152 | 0.431 | 8.62E-09 |
| OR10Z1 | rs857685 | A/C | IEpoolvsP | C | 0.09783 | 0.4138 | 2.71E-07 |
