# Supplementary material for: Combined genetic effects of EGLN1 and VWF modulate thrombotic outcome in hypoxia revealed by Ayurgenomics approach
Source: J Transl Med. 2015 Jun 6;13:184. doi: 10.1186/s12967-015-0542-9 (PMC4457985; doi:10.1186/s12967-015-0542-9)
Supplement: Additional file 6: — Association of SNPs varying between Prakriti with selection pressures such as climatic conditions, mode of subsistence, pathogen pressure and cultural practices (source: http://genapps2.uchicago.edu:8081/dbcline/). [file 12967_2015_542_MOESM6_ESM.doc]

| Gene | Query SNP | dbCline SNP | Global Der. Freq. | Environmental Variable | Bayes Factor | Transformed Rank |
| --- | --- | --- | --- | --- | --- | --- |
| LEPR | rs1171271 | rs6588147,rs10889558, rs1137100,rs11208677 | 0.37,0.39,0.31,0.36 | Latitude | 2.753E1,1.795E2,2.086E1,2.280E1 | 0.02,0.01,0.03,0.03 |
|  |  | rs6588147,rs1327121, rs10889558,rs1782754, rs2154381,rs1171269, rs1022981,rs1137100, rs11208677,rs12028951 | 0.37,0.37,0.39,0.34,0.63,0.33,0.33,0.31,0.36,0.36 | Absolute Latitude | 1.496E2,1.657E1,2.172E1,9.566E0,1.092E1,2.639E1,1.113E1,3.208E1,1.910E1,3.410E1 | 0.01,0.04,0.03,0.04,0.05,0.02,0.04,0.02,0.03,0.02 |
|  |  | rs6588147 | 0.37 | Minimum Temperature  (Winter) | 2.614E1 | 0.01 |
|  |  | rs6588147,rs1327121, rs2154381 | 0.37,0.37,0.63 | Maximum Temperature (Summer) | 3.245E0,3.488E0,3.192E0 | 0.03,0.03,0.04 |
|  |  | rs6588147,rs12409877, rs1327121,rs10889558, rs1782754,rs2154381, rs1171269,rs1022981, rs1137100,rs11208677, rs12028951 | 0.37,0.44,0.37,0.39,0.34,0.63,0.33,0.33,0.31,0.36,0.36 | Short Wave Radiation Flux  (Winter) | 8.506E1,5.770E1,6.078E2,4.933E1,1.010E1,8.937E1,4.155E2,6.766E1,2.547E1,2.445E2,7.308E1 | 0.01,0.01,0.004,0.02,0.04,0.01,0.002,0.01,0.02,0.01,0.01 |
|  |  | rs1782754,rs2154381, rs11208677,rs12028951 | 0.34,0.63,0.36,0.36 | Relative Humidity (Winter) | 4.665E0,4.074E2,3.758E1,3.678E0 | 0.02,0.001,0.003,0.03 |
| OR6K3 | rs857703 | rs857703 | 0.29 | Horticulture | 4.822E0 | 0.02 |
|  |  |  |  | Intensive Agriculture | 1.532E0 | 0.03 |
| UCP2 | rs659366 | rs659366 | 0.37 | Intensive Agriculture | 4.735E2 | 0.001 |
|  |  |  |  | Cereals | 2.718E4 | 0.001 |
|  |  |  |  | Fat/Meat/Milk | 1.334E0 | 0.04 |
|  | rs660339 | rs660339 | 0.38 | Short Wave Radiation Flux  (Winter) | 9.134E0 | 0.04 |
|  |  |  |  | Humid Temperate | 1.450E0 | 0.05 |
|  |  |  |  | Intensive Agriculture | 2.173E0 | 0.03 |
|  |  |  |  | Cereals | 3.962E0 | 0.03 |
| OLR1 | rs3741860 | rs3741860 | 0.39 | Horticulture | 2.639E0 | 0.04 |
| VWF | rs1063856 | rs1063856 | 0.73 | Short Wave Radiation Flux  (Summer) | 1.563E1 | 0.01 |
|  |  |  |  | Dry | 1.218E0 | 0.04 |
| SPTA1 | rs857691 | rs857691 | 0.58 | Absolute Latitude | 3.154E1 | 0.02 |
|  |  |  |  | Precipitation Rate (Summer) | 2.000E0 | 0.05 |
|  |  |  |  | Horticulture | 8.418E2 | 0.002 |
|  |  |  |  | Intensive Agriculture | 1.601E4 | 0.0004 |
|  |  |  |  | Cereals | 5.617E1 | 0.01 |
|  |  |  |  | Roots/Tubers | 1.918E1 | 0.01 |
| SPTA1, OR10Z1 | rs857721,  rs857685 | rs857691 | 0.58 | Absolute Latitude | 3.154E1 | 0.02 |
|  |  | rs857725,rs857691 | 0.32, 0.58 | Cereals | 9.048E0,5.617E1 | 0.02, 0.01 |
|  |  | rs857725 | 0.32 | Foraging | 3.433E0 | 0.05 |
|  |  | rs2518491,rs857691,rs2518493 | 0.29,0.58,0.28 | Horticulture | 1.183E2,8.418E2,1.475E0 | 0.003,0.002,0.05 |
|  |  | rs2479868,rs2518491,rs857725,rs857691,rs2518493,rs12041363 | 0.32,0.29,0.32,0.58,0.28,0.29 | Intensive Agriculture | 2.232E0,7.568E3,1.396E1,1.601E4,8.083E0,2.257E0 | 0.03,0.001,0.01,0.0004,0.01,0.03 |
|  |  | rs857725 | 0.32 | Longitude | 3.314E0 | 0.02 |
|  |  | rs857691,rs2518493 | 0.58,0.28 | Precipitation Rate (Summer) | 2.000E0,8.531E0 | 0.05,0.01 |
|  |  | rs2518491 | 0.29 | Precipitation Rate (Winter) | 1.63E+00 | 0.05 |
|  |  | rs2518491,rs857725,rs2518493 | 0.29,0.32,0.28 | Relative Humidity (Winter) | 9.644E0,1.061E1,2.615E0 | 0.01,0.01,0.03 |
|  |  | rs857691 | 0.58 | Roots/Tubers | 1.918E1 | 0.01 |
